# Effect of Flexibility Training Associated with Multicomponent Training on Posture and Quality of Movement in Physically Inactive Older Women: A Randomized Study

**DOI:** 10.3390/ijerph182010709

**Published:** 2021-10-13

**Authors:** Andressa Crystine da Silva Sobrinho, Mariana Luciano de Almeida, Guilherme da Silva Rodrigues, Larissa Chacon Finzeto, Vagner Ramon Rodrigues Silva, Rodrigo Fenner Bernatti, Carlos Roberto Bueno Junior

**Affiliations:** 1 School of Medicine of Ribeirao Preto, University of Sao Paulo (USP), Avenida Bandeirantes 3900, Ribeirao Preto 14049-900, SP, Brazil; andressa.sobrinho@usp.br (A.C.d.S.S.); guirodrigues@usp.br (G.d.S.R.); 2 College of Nursing of Ribeirao Preto, University of Sao Paulo (USP), Avenida Bandeirantes 3900, Ribeirao Preto 14049-900, SP, Brazil; ml.almeida@usp.br; 3 School of Physical Education and Sport of Ribeirao Preto, University of Sao Paulo (USP), Avenida Bandeirantes 3900, Ribeirao Preto 14049-900, SP, Brazil; larissa.finzeto@usp.br; 4 School of Applied Sciences, University of Campinas (UNICAMP), Rua Pedro Zaccaria 1300, Limeira 13484-350, SP, Brazil; silvavagnerramon@gmail.com; 5 University of Franca (UNIFRAN), Avenida Dr. Armando de Sáles Oliveira 201, Franca 14404-600, SP, Brazil; rodrigo_fenner@yahoo.com

**Keywords:** postural stability, body balance, physical activity, biodynamic response, aging

## Abstract

Background: Multicomponent training has considerable adherence among older populations, but there is a lack of literature on the benefits of this training on older people’s posture. Literature also lacks stretching protocols that work the body in an integrated/unified way and respect the principle of individuality in exercise training. We evaluated the effect of a multicomponent training protocol combined or not with flexibility training in improving the posture and quality of movement in physically inactive older women, according to a score lower than 9.11 in the Modified Baecke Questionnaire for the Elderly (MBQE). Methods: 142 participants were evaluated and randomized in three training groups: multicomponent training (MT = 52), multicomponent and flexibility training (MFT = 43), and a control group (CG = 47). We evaluated joint amplitude using goniometry, flexibility with sit and reach and hands behind the back tests, quality of movement with the functional movement screen, and posture using biophotogammetry. Results: The MFT group had 15 parameters—flexibility and posture—with a very large effect size (ES > 1.30) and nine with average ES (0.50–0.79). MT presented two variables with large ES (0.80–1.25) and seven with average ES. CG presented three variables with high ES and five with average ES. Both interventions improved the quality of movement. Conclusions: These results demonstrate that 14 weeks of multicomponent and flexibility training in a group intervention can improve flexibility and posture levels in physically inactive older women.

## 1. Introduction

Alterations in postural pattern constitute one of the changes that most affect older adults, especially women, and derive from a set of factors that include hormonal alterations, sarcopenia, osteopenia, a reduction in the level of physical activity, and tendon and ligament fragility [1,2]. Postural imbalances are also factors that contribute to an increase in the incidence of falls [3,4], and are associated with back problems [2]. Back problems are the second biggest public health service complaint among the middle-aged and older population [5,6].

Motor performance is a combination of physical fitness and quality of movement. It involves performing specific actions with less energy expenditure, greater ease, and lower muscle recruitment [7]. Quality of movement has been associated with cognitive function, functional capacity, and the ability to perform day-to-day activities [8]. The functional movement screen (FMS) is a new instrument that aims to analyze seven global and universal basic patterns of movement, resulting in a score for each person’s quality of movement [9].

The literature has exhaustively demonstrated the benefits of strength training and cardiorespiratory function in mortality, morbidity, and quality of life analyses for older adults [10,11]. In turn, the benefits associated with flexibility training are not clear in the literature, which can even be observed in the positioning of the American College of Sports Medicine (ACSM), regarding physical exercise for the older adults [10]. Until now, the beneficial effects of flexibility have only been demonstrated in specific situations, such as an improvement in musculoskeletal discomforts of office workers [12], quality of sleep in chronic insomnia patients [13], and mechanical properties of spastic muscle in patients with chronic stroke [14], as well as being in scientific articles with no great international impact, so that prescribing them indiscriminately does not appear to be a practice based on scientific evidence.

Multicomponent training is a modality that features the characteristic of embracing different physical capacities in the same session, promoting a global improvement in the general state of health of older people [15]. One of the challenges for the older population to remain active is adhesion to physical exercise programs—multicomponent training shows high adhesion and adherence rates and can be carried out in groups, which favors socialization among participants [16]. It has been demonstrated that multicomponent training promotes an improvement in flexibility [16,17].

The ACSM points out that there is still a lack of evidence about flexibility and its functionality in the elderly, and in its exercise guidelines for the elderly. In addition to not knowing for certain which is the best protocol for the development of this physical capacity, studies usually present their evaluation through the sit and stand test, a test to evaluate the hamstrings [17]. Furthermore, the literature shows that resistance training has been shown to improve flexibility both in young people [18] and in older people [19] when performed with a full range of motion. Therefore, it is also questionable whether specific flexibility training is necessary [20,21,22].

However, the effect of multicomponent training on body posture is unknown, as well as its effect, in association or not with flexibility training, on quality of movement. Within this context, this study aimed to evaluate the effect of a multicomponent training protocol combined or not with flexibility training on the postural evaluations and quality of movement of physically inactive older women. The study hypothesis was that the older women in the multicomponent training group with an emphasis on flexibility would present better improvements than the women from the other two groups and that the multicomponent training group would present better results than the control group.

## 2. Materials and Methods

### 2.1. Study Design

The participants were recruited by advertising in local media and on social media. Before the first assessment, the participants were invited to a presentation meeting, where they received information on the research objective and details about the test protocol—they also signed the informed consent form at the end of the session. After this stage, the participants were divided into two groups, separated by age into 60 to 65 and 66 to 70 years old. After recruitment, a blinded researcher randomly divided the participants into three groups using the random number generator tool of the Microsoft Excel^®^ version 2013: multicomponent training (MT), multicomponent plus flexibility training (MFT), and control group (CG), guaranteeing the homogeneity between the groups in relation to age. In the power analysis, 40 participants per group would be necessary to detect a difference between means of 8.5 cm for the primary outcome (frontal plane asymmetry or sagittal plane asymmetry), with the alpha error probability set at 0.05 and power adjusted to 0.8. We used G Power 3.1.9.7 to calculate the power.

**Figure 1:**
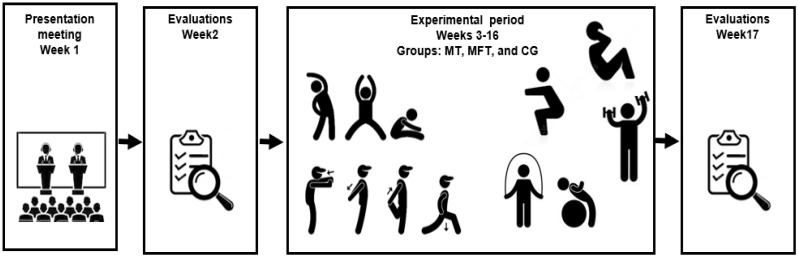
Study design. CG; control group; MFT, multicomponent and flexibility training group; MT, multicomponent training.

The participants in the control group were told not to engage in physical exercise during the entire duration of the study. Pre and post experimental period evaluations were conducted. The experiment lasted 14 weeks (Figure 1).

### 2.2. Participants

The inclusion criteria adopted were women aged between 60 and 70, with a medical certificate releasing them to practice physical activity, and who were physically inactive according to a score lower than 9.11 in the Modified Baecke Questionnaire for the Elderly (MBQE) [23]. The exclusion criteria were having diseases and/or functional limitations (motor, auditory, and visual disorders) that would impede carrying out the tests and the physical training proposed, and absences in more than 25% of the physical training sessions.

The research and the informed consent form were submitted to and approved by the Ethics Committee for Research on Human Beings of the School of Physical Education and Sport of Ribeirão Preto of the University of São Paulo (CAAE:63681517.3.0000.5659) and registered in the Brazilian Register of Clinical Trials (REBEC: RBR-8hqwmx).

### 2.3. Interventions

#### 2.3.1. Multicomponent Training

The multicomponent training was constituted of two 90-min classes per week, divided into an initial 15 min of warm-up, balance, motor coordination, and games, 35 min of muscle strength, 35 min of aerobic activities, and a final five minutes of relaxation, with the aim of developing coordination motor abilities and conditioning motor abilities [15,20]. The intensity of the training was monitored using the Borg scale, with the aim of perceiving effort in values from three to 10, progressively, on a scale from zero to 10 (weeks 1 to 2: 3 to 4; weeks 3 to 5: 4 to 6; weeks 6 to 8: 6 to 7; weeks 9 to 11: 7 to 8; weeks 11 to 14: 8 to 10), representing moderate to high intensity physical exercise [21]. The training focused on strengthening the following muscles: rectus abdominal muscle, abdominal external oblique muscle, abdominal internal oblique muscle, transverse abdominal muscle, gluteus, abductors, knee flexors and extensors, deep neck flexors, serratus, rhomboid, middle and ascending trapezius, rotator cuff, and paravertebral or spinal erectors.

A team of trained exercise professionals supervised the multicomponent training. Muscle strength and aerobic activities were carried out in the form of a circuit, using basic exercises of pulling, pushing, squatting, lifting, and holding—which strengthens the regions mentioned above—such as squats, different formats of displacement (lateral, side, front, back, with high and low knees), pelvic elevation, sinks, curved row, and reverse crucifix, etc. In each training session, ten exercises were used, with 2 min of execution and 1 min of rest between exercises. The circuit was performed twice with a 7-min water break between sets.

#### 2.3.2. Individualized Flexibility Training

Flexibility capacity was trained using the active stretching with accessories method, following the protocol proposed by Nelson and Kokkonen [18], who follow the recommendations of the American College of Sports Medicine [22] in relation to volume and intensity. The participants were separated into groups for stretching geared toward postural alterations (hip flexor muscles, spinal extensors, scapula elevators, and protractors), focusing on individual needs, identified after carrying out a postural analysis. The exercise protocol contained for each postural compensation four exercise complexity levels, adding a new complexity every four weeks after two weeks in the level 1. The intensity and volume protocol were divided into four levels with stretching time progression and pain perception using the pain scale. The training was carried out twice a week (Table 1).

### 2.4. Evaluations

For the participant characterization, a questionnaire was used, elaborated by the researchers to analyze demographic and health data. Blood pressure was measured using an automatic digital arm pressure measurer (OMRON^®^, HEM-7113 model, Songjiang, China), as well as conducting an anthropometry analysis [body mass, height, hip and waist circumference, and body mass index (BMI)], and fat percentage was measured using bioelectrical impedance (BIA, Maltron BF-906^®^ model, Rayleigh, UK). The participants’ physical activity level was measured subjectively using the MBQE [23], together with a triaxial accelerometer (GT3X-BT from ActiGraph, Pensacola, FL, USA). The participants were instructed to use it for a week, but four days a week and one day of the weekend were considered for the calculation, with the intensities of the activities being stipulated by Freedson et al. [24].

#### Motor Evaluations and Evaluative Instruments

Flexibility was analyzed by the hands behind the back and sit and reach tests, from the battery of tests of Rikli and Jones [25]. The analysis of the most common postural alterations and identification of the degree of accentuation were carried out via biophotogammetry, a test that uses photographs to measure the degree of postural deviations in each structure of the body. To obtain the images, a digital photographic camera was positioned on a leveled and plumbed tripod at a height of 95 cm in relation to the floor and at a distance of three meters from the participant; this being the best position suggested by the literature [26,27]. A plumb line with two polystyrene balls separated by a meter’s distance was placed beside the participant. This distance was used as a calibrator, as according to the protocol of the SAPO^®^ program. The protocol has a total of 32 points, which can be analyzed in the different photographic views [26].

To measure the older women’s joint movement amplitude, a goniometer was used, which analyzes joint flexibility [28,29]. To evaluate quality of movement, muscle asymmetries, and risk of injury due to postural modifications, a functional evaluation test was used that relates balance, strength, and muscle-tendon amplitude: the functional movement screen (FMS). This instrument aims to determine mobility and stability problems, identifying weak points in individuals who seek to maintain or raise their physical activity levels [9].

### 2.5. Statistical Analysis

The data obtained were organized in a double-entry database, using the Excel^®^ (Microsoft Corporation, Redmond, WA, USA) program version 2013 and the SPSS^®^ program version 20.0 (International Business Machines, Armonk, NY, USA). The data were presented with means and standard deviations. To evaluate the data normality the Kolmogorov-Smirnov was used, and the variances were analyzed by the Levene test. The analysis of the training comparisons was conducted using the two-way ANOVA statistical method of repeated measures. The ANOVA for repeated measures observes levels of comparison between groups and variables, namely: time effect (evaluates the variables in 1 factor—time, pre versus post) and the interaction between group and time (which evaluates the variables in two simultaneous factors between groups and times) with Tukey’s post-hoc test. For all statistical analyses, it was considered as an independent variables time and group. As dependent variables, the joint range of motion (goniometry), flexibility (sit and reach test and hand on the back), postural analysis (biophotogametry), and quality of the movement (FMS) are examples. The effect size, a descriptive statistic that serves as a complement to the statistical significance test, was calculated using Cohen’s d, where values from 0.50 to 0.79 represent an average effect, values between 0.80 and 1.29 is a large effect, and more than 1.30 is a very large effect—numbers below 0.50 were considered a small effect [30]. The significance level considered was 5%.

## 3. Results

**Figure 2:**
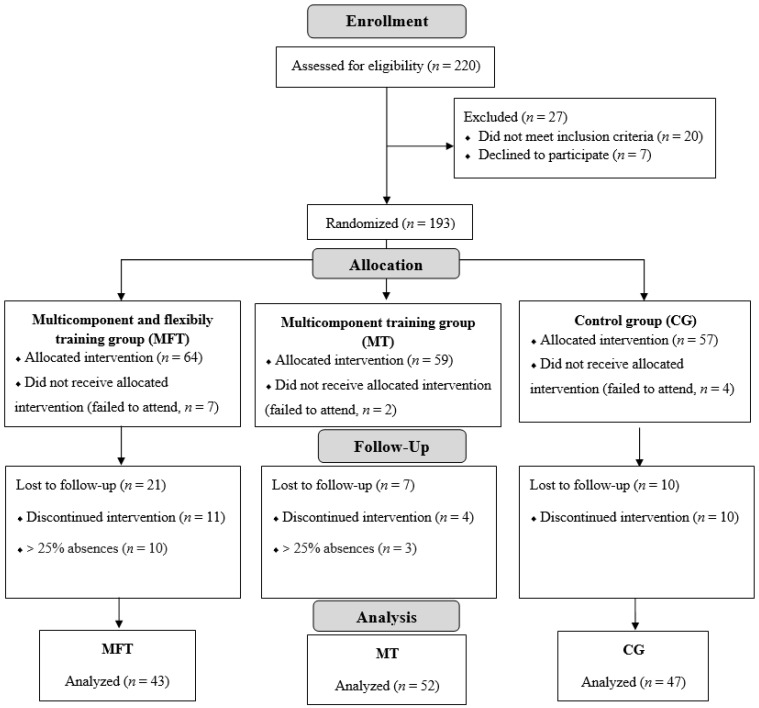
CONSORT flow diagram.

In the analyses, 43 women were included in the MFT group, 52 in the MT group, and 47 in the CG, as presented in Figure 2. There was no statistical difference between the groups in the means for age (63.4 ± 5.6, considering all groups) and age at the last menstrual cycle (48.1 ± 5.8, considering all groups) (Table 2). A time effect is noted in the body mass variable (F = 8.131; *p* = 0.006), which after the intervention was lower than the baseline, and in the physical activity level evaluated by the questionnaire (F = 4.201; *p* = 0.010), with an increase between the pre- to the post-intervention moments.

There was a group and time interaction in the fat percentage variable (F = 5.006; *p* = 0.029) and physical activity level evaluated using accelerometry (F = 3.781; *p* = 0.016), observing an improvement from the pre- to the post-moments in both variables only in the MFT group—the MT group presented an improvement in the accelerometry between both evaluations. There was also a group and time interaction for the SBP (F = 3.095; *p* = 0.035) and DBP (F = 13.729; *p* < 0.001) variables, with a reduction in the SBP observed only in the MFT group—the DBP varied only in the CG, presenting an increase from the pre- to the post-experimental period moments. We also observed this interaction for waist circumference (F = 10.027; *p* = 0.003), with lower results in the CG and MFT groups in relation the MT group in the post moment. Finally, a time and group interaction was also observed in the BMI variable (F = 17.67; *p* = 0.048), with a deterioration of the CG between the pre- and post-intervention moments (Table 2).

In Table 3 it is possible to observe the time effect on the sit and reach (F = 51.59; *p* < 0.001), shoulder extension (F = 69.81; *p* < 0.001), knee extension (F = 12.08; *p* = 0.001), and knee flexion (F = 12.08; *p* = 0.001) variables, with an improvement in these parameters at the post-intervention moment in relation to the baseline. It was possible to observe an improvement from the pre- to the post-intervention moments in the sit and reach test—and deterioration in the other three variables. There was group and time interaction in the functional movement screen variable (F = 7.15; *p* = 0.001), with an improvement between the moments in the MT and MFT groups—and the CG presented lower results than the MT group at the post-intervention moment. There was group and time interaction for the goniometry in the following variables: cervical flexion (F = 2.42; *p* < 0.001), hip extension (F = 7.87; *p* = 0.001), hip flexion (F = 3.45; *p* = 0.034), cervical extension (F = 17.57; *p* < 0.001), shoulder flexion (F = 9.97; *p* < 0.001), lumbar extension (F = 3.42; *p* = 0.035), lumbar flexion (F = 15.32; *p* < 0.001), ankle flexion (F = 3.57; *p* = 0.031), and ankle extension (F = 3.57; *p* = 0.031). Of these nine variables, there was an improvement from the pre- to the post-intervention moments for everyone in the MFT group, five in the MT group, and three in the CG—these results demonstrate that the MFT group was better than the MT and CG groups in terms of gaining joint movement amplitude (Table 3).

In Supplementary Table S1 we observe that in the postural characterization of the anterior view there was a time effect on the limb length difference (LLD)—(F = 52.94; *p* < 0.001), tibial tuberosity alignment (TTA)—(F = 32.70; *p* < 0.001), horizontal acromion alignment (HAA)—(F = 106.40; *p* < 0.001), right Q angle (RQA)—(F = 130.32; *p* < 0.001), left Q angle (LQA)—(F = 83.46; *p* < 0.001), and horizontal head alignment (HHA)—(F = 149.19; *p* < 0.001), with a post-intervention improvement in relation to the baseline.

Supplementary Table S2 describes the data relating to the posterior view of the postural analysis, demonstrating a time effect on the horizontal scapular asymmetry (HSA)—(F = 54.09; *p* < 0.001), right leg and hindfoot angle (RLHA)—(F = 17.57; *p* < 0.001), and left leg and hindfoot angle (LLHA)—(F = 29.90; *p* < 0.001), with an improvement in postural adjustment from the pre-to the post-intervention moments.

The time effect was also observed in Supplementary Table S3 relating to the right lateral view of the postural analysis in the vertical torso alignment (VTA)—(F = 30.69; *p* < 0.001), vertical alignment of the head with the acromion (VAHA)—(F = 19.30; *p* < 0.001), hip alignment (HA)—(F = 33.57; *p* < 0.001), and horizontal pelvic alignment (HPA)—(F = 75.63; *p* < 0.001), which after the intervention had better results in relation to the baseline. There was group and time interaction in the horizontal alignment of the head with the C7 (HAHC7)—(F = 201.29; *p* = 0.03) and ankle angle (AA)—(F = 211.87; *p* = 0.001) variables—at the post-moment both intervention groups presented an improvement in relation to the pre-intervention moment. In both variables the MFT group presented better results in relation to the MT group, at the post-intervention moment.

Supplementary Table S4 presents the postural analysis in the left lateral view, which presented a time effect on the variables vertical alignment of the head with the acromion (VAHA)—(F = 39.37; *p* < 0.001), vertical body alignment (VBA)—(F = 136.70; *p* < 0.001), hip alignment (HA)—(F = 25.02; *p* < 0.001), vertical torso alignment (VTA)—(F = 39.56; *p* < 0.001), and knee angle (KA)—(F = 5.29; *p* = 0.006), with an improvement from the pre- to the post-intervention moment. Moreover, we observed time and group interaction in the horizontal alignment of the head with the C7 (HAHC7)—(F = 5.15; *p* < 0.007) and ankle angle (AA) (F = 6.93; *p* = 0.001) variables. In both variables, the MFT group presented better results and the CG worse results in relation to the MT group, at the post-intervention moment. Both intervention groups demonstrated an improvement from the pre- to the post-experimental moment in the HAHC7 and AA variables.

Supplementary Table S5 shows it is possible to note time and group interaction in the frontal plane asymmetry (FPA)—(F = 14.56, *p* < 0.001) and sagittal plane asymmetry (SPA)—(F = 11.76, *p* < 0.001) variables—the MT and MFT groups presented an improvement in the results at the post moment in relation to the pre-intervention moment.

In Table 4, there is a summary of all variables related to postural evaluation (biophotogammetry) based on their effect sizes.

Concerning the analysis of the effect size in the whole set of variables studied, the MT group presented an improvement in nine variables with at least a medium effect size, where two variables had a large effect, and seven had a medium effect. In the MFT group, this number was 24, of which 15 variables had a very large effect size and nine had a medium effect size. In the control group, there was an improvement in eight variables with at least a medium effect size - three of them with a large effect and five with a medium effect. Considering only postural variables, the number of variables was four, one, and three for the MT group; sixteen, nine, and seven for the MFT group; five, one, and four for the CG group.

Finally, in terms of the effect size, it was also possible to observe that the MFT group resulted in a better realignment of the center of mass, adjusting the body structures both on the sagittal plane and on the frontal plane (Supplementary Table S5).

## 4. Discussion

The findings showed the importance of combining multicomponent training with flexibility training and its effects in terms of a global improvement in postural deviations, movement amplitude, and quality of movement in older women, which have not yet been described in the literature. However, these results are consistent with previous studies that have used different training programs, such as yoga, global postural reeducation (GPR), pilates, and physiotherapy sessions, with separate analyses of postural and flexibility parameters in individualized regions, such as in the hip, hamstring, shoulder, and spine [19,31].

The present study contained a progression of complexity of exercises and tension time (time remaining in the greatest movement amplitude), per exercise, with a weekly dose of 40 min to one hour and 40 min, and with a 14-week intervention duration. The studies with middle-aged adults present a duration of only 30 s of tension for exercises, with no progression of complexity and tension time, starting from one to three weekly sessions during eight to 10 weeks [32]. The intervention designs with flexibility result in a weekly dose of 1200 s per training session, 50% lower than prescribed in the beginning of the present intervention in the MFT group, and a shorter period, lasting from two to six weeks of intervention [32].

MFT was the group that presented more gain in the flexibility tests compared with the other groups. It was possible to note a moderate to strong effect size with the flexibility training, mainly on the more complex joints of the body, such as the ankle, hip, and shoulder. These same regions present the lowest range of motion (ROM) due to the biarticular characteristic of the muscles and reduced levels of physical activity, which promote muscle shortening, where the hip and ankle joints stand out as a factor for an increase in risks of falls [33].

All the participants in our study presented a lower ankle ROM than necessary for the adequate ROM required at the start of the training protocol adopted in this study. The literature shows that both static and dynamic stretching exercises help in improving ankle ROM, and this is important as its reduction is a well-established process in aging [29]. Studies have shown that a reduced hip ROM leads to lower speed of movement of the lower limbs, making balance recovery strategies, such as the step, even more difficult, and increasing the chances of falls in older women [33,34].

It has been also demonstrated that a reduction in shoulder pain through training protocols, including stretching exercises in the training sessions and causing an improvement in shoulder ROM, was directly related with stability and pain [32]. Moreover, other studies with older women have shown an association between greater ROM, an increase in daily life activities, and quality of life [34,35]. The result obtained by our study showed that the MT group obtained a greater effect on quality of movement, measured by the FMS effect size, than the MFT group, even though the latter presented a functional improvement, which could contribute to better performance in daily life activities (DLAs).

Moreover, the values obtained in the MT and MFT groups are higher than those presented in the literature. The study by Mitchell et al. [36] reported a total score of 12.2 points in a study with 97 older women with a mean age of 65.7. Perry and Koehle [37] evaluated 12 women older than 65, who obtained a total score of 13.17 points, showing that even though they are physically inactive, the older women in our study are above the mean listed in the literature.

The MT group presented an improvement in four variables related to postural alignment, and the MFT group an improvement in 16 variables. The postural evaluation showed significant differences in the regions that present complex joints, such as the scapular girdle, the pelvic girdle, and ankle between the groups, as was observed in the ROM test, improving the alignment of body structures. The MFT group presented a greater effect size in post-intervention tests, which corroborates with the data from the study by Bandeira et al. [38] Their results showed that physically active older adults presented fewer changes in the curvature of the thoracic spine than sedentary participants. When individuals present a greater pattern of postural deviations, they also show a greater body oscillation, leading to a rise in falls, injuries, and an increase in public healthcare costs [39]. The study of Ishikawa et al. [40] showed that the loss of angulation of the curvature of the lumbar lordosis, a common alteration in older women, increased postural instability and led to a greater chance of falls due to the displacement of the gravitational line on the sagittal plane [41].

Regarding systolic blood pressure, only the MFT group showed improvement in this variable, demonstrating that flexibility training can contribute to this adaptation. Benefits of flexibility training for health variables are not yet evident in the literature. Yamamoto et al. [42] carried out a cross-sectional study to test the hypothesis that a less flexible body would have greater arterial stiffness. They studied people aged 20 to 83 years; using the sit-and-reach test, they concluded that worse flexibility is associated with greater arterial stiffness in people over 40, regardless of cardiorespiratory fitness and muscle strength. The authors suggest that flexibility may be a predictor of arterial stiffness, as in the study by Nishiwaki et al. [43] The latter conducted an observational cross-sectional study with 1150 adults, aged between 18 and 89 years. In addition, Nishiwaki et al. [44] showed an association between higher levels of flexibility and lower arterial stiffness, but a reduction in arterial stiffness caused by four weeks of flexibility training was not significantly correlated with increased flexibility. Thus, our results help support the hypothesis that flexibility training can contribute to improving other health parameters, such as blood pressure. We have presented this effect on blood pressure for the first time since these previous studies have demonstrated it on arterial stiffness.

### Limitation

This study presents strengths, but it is necessary to highlight some limitations, such as the time established for the training could have been longer. However, 14 weeks is sufficient to induce adaptations in physical capacities by physical training, even more in physically inactive individuals.

## 5. Conclusions

Multicomponent training associated with a flexibility training protocol based on a group intervention proved to be more efficient for postural deviations and equally effective in the quality of movement of physically inactive older women when compared to multicomponent training.

## Figures and Tables

**Table 1:** Flexibility training protocol adopted in the intervention.

	Level 1	Level 2	Level 3	Level 4
Week of intervention	1–2	3–6	7–10	11–14
Duration of session	20′	30′	40′	50′
Time under tension	10″	15″	20″	25″
Interval between series	10″	15″	20″	25″
Series per exercise	2	3	4	5
Pain level *	1 a 3	2 a 4	4 a 6	6 a 8
Exercises per body region ^$^	2	3	3	4
Weekly dose ^#^	2400″	3600″	4800″	6000″

Note: *—numeric visual/verbal scale of pain from 0 to 10; ^$^—8 body regions were worked in each individual—initial evaluations of each participant were considered for choice of these regions; ^#^—weekly dose (seconds) = duration of session (min) × 2 (sessions/week) × 60 (seconds/minute).

**Table 2:** Characterization of the participants at the pre and post experimental period moments.

	MT (*n* = 52)			MFT (*n* = 43)			CG (*n* = 47)		
	Pre	Post	Effect Size	Pre	Post	Effect Size	Pre	Post	Effect Size
Age (years)	63.1 ± 5.5			63.4 ± 5.6			64.0±4.9		
Last menstrual cycle (years)	47.5 ± 6.3			48.1 ± 5.8			48.0 ± 6.2		
Height (m)	1.56 ± 7.0			1.59 ± 6.9			1.58 ± 7.80		
Body mass (kg) ^‡^	75.1 ± 14.4	73.8 ± 12.8	−0.096	74.7 ± 16.0	72.1 ± 15.8	−0.164	76.1 ± 18.8	79.3 ± 10.0	0.222
BMI (kg/m^2^) ^†^	29.4 ± 5.0	29.2 ± 4.7	−0.041	28.4 ± 4.7	27.9 ± 5.3	−0.100	25.3 ± 2.7	28.4 ± 4.1 ^$,#,^*	**0.912**
SBP (mmHg) ^†^	131.9 ± 21.3	129.0 ± 10.2	−0.184	129.2 ± 18.8	123.5 ± 15.4 *	−0.333	135.6 ± 17.9	136.5 ± 12.4 ^#^	0.059
DBP (mmHg) ^†^	76.8 ± 10.6	76.8 ± 8.3	0.000	76.6 ± 10.6	71.9 ± 10.0	−0.456	73.8 ± 10.1	79.1 ± 6.3 ^#,^*	**0.646**
WC (cm) ^†^	100.5 ± 11.3	100.1 ± 9.2	−0.039	96.8 ± 12.0	94.3 ± 12.1 ^$^	−0.207	97.4 ± 12.9	98.2 ± 9.9 ^$^	0.070
HC (cm)	107.5 ± 12.3	106.1 ± 9.2	−0.130	106.1 ± 9.7	105.1 ± 10.5	−0.099	107.0 ± 10.7	104.6 ± 9.2	−0.241
BF (%) ^†^	41.2 ± 8.1	40.1 ± 6.8	−0.148	46.2 ± 6.9	35.8 ± 7.9 *^,$^	**−1.405**	43.1 ± 6.9	46.3 ± 6.7 ^$,#,^*	0.471
MBQE (scores) ^‡^	6.9 ± 5.3	11.7 ± 7.5	**0.750**	6.3 ± 5.4	12.6 ± 5.0	**1.212**	6.4 ± 5.0	8.6 ± 7.6	0.349
PA (accelerometer, counts) ^†^	382 ± 1361	877 ± 401 *	**0.562**	386 ± 176	961 ± 401 *^,$^	**1.993**	348 ± 171	592 ± 365 ^#^	**0.911**

Note: MT, multicomponent training; MFT, multicomponent and flexibility training; CG, control group; BMI, body mass index; SBP, systolic blood pressure; DBP, diastolic blood pressure; WC, waist circumference; HC, hip circumference; BF, body fat; MBQE, Modified Baecke Questionnaire for the Elderly; PA, physical activity. ^†^ Interaction between time and group (*p* < 0.05). ^‡^ Time effect (*p* < 0.05). * *p* < 0.05 in relation to the pre-intervention moment in the same group. ^#^ *p* < 0.05 in relation to the MFT at the same moment. ^$^ *p* < 0.05 in relation to the MT at the same moment. Bold: effect size ≥ 0.50.

**Table 3:** Flexibility and movement quality at the pre and post experimental period moments.

	MT (*n* = 52)			MFT (*n* = 43)			CG (*n* = 47)		
	Pre	Post	Effect Size	Pre	Post	Effect Size	Pre	Post	Effect Size
Hands behind the back (cm)	−9.5 ± 9.7	−7.6 ± 9.6	0.197	−5.0 ± 11.8	−4.1 ± 9.2	0.086	−7.6 ± 10.9	−7.8 ± 11.9	−0.018
Sit and reach (cm) ^‡^	−3.1 ± 8.8	2.0 ± 7.6	**0.626**	1.1 ± 9.9	3.3 ± 10.0	0.213	−1.9 ± 7.9	−2.5 ± 4.9	−0.094
Goniometry (°)									
Cervical extension ^†^	14.2 ± 5.4(38)	15.7 ± 6.8 *	0.246	15.7 ± 7.6(46)	18.3 ± 7.5 *	0.344	12.6 ± 6.4(47)	12.6 ± 7.7 ^#^	0.000
Cervical flexion ^†^	34.9 ± 14.4(38)	36.6 ± 14.8	0.116	33.8 ± 12.1(46)	37.3 ± 13.2 *	0.277	31.4 ± 12.0(47)	30.6 ± 11.8 *^,#,$^	−0.067
Shoulder extension ^‡^	30.3 ± 8.3	30.6 ± 8.6	0.036	30.4 ± 9.2	31.6 ±10.2	0.124	27.1 ± 9.7	30.2 ± 8.7	0.337
Shoulder flexion ^†^	156.8 ± 19.4	156.9 ± 21.6	0.005	146.7 ± 18.2	148.4 ± 21.0 *	0.087	137.6 ± 29.9	141.3 ± 30.0 *^,#^	−0.124
Lumbar extension ^†^	15.5 ± 7.3	16.8 ± 8.3	0.167	16.7 ± 9.6	19.4 ± 10.6 *^,$^	0.267	16.6 ± 7.7	19.6 ± 8.6 *^,$^	0.368
Lumbar flexion ^†^	73.2 ± 10.1	73.3 ± 9.9	0.010	75.7 ± 9.6	77.7 ± 11.4 *^,$^	0.190	74.3 ± 11.3	78.6 ± 11.2 *^,$^	0.382
Hip extension ^†^	4.7 ± 1.7(38)	5.8 ± 3.9 *	0.393	4.5 ± 1.8(40)	6.9 ± 1.8 *	**1.333**	12.6 ± 6.4(47)	12.6 ± 7.7 ^$,#^	0.000
Hip flexion ^†^	58.0 ± 17.2(38)	69.2 ± 17.4 *	**0.647**	62.4 ± 10.3(40)	71.1 ± 10.0 *	**0.857**	31.4 ± 12.0(47)	30.6 ± 11.8 ^$,#^	−0.067
Knee extension ^‡^	87.7 ± 20.8	86.9 ± 20.4	−0.039	93. 8 ± 20.4	96.5 ± 19.1	0.137	94.8 ± 16.3	96.1 ± 19.2	0.073
Knee flexion ^‡^	87.7 ± 20.8	86.9 ± 20.4	−0.039	93.8 ± 20.4	96.5 ± 19.1	0.137	94.8 ± 16.3	96.1 ± 19.2	0.073
Ankle extension ^†^	8.7 ± 3.2(38)	9.7 ± 4.8 *	0.250	9.6 ± 2.9(47)	12.8 ± 3.1 *^,$^	**1.067**	8.7 ± 2.9(47)	7.2 ± 3.7 ^$,^	−0.455
Ankle flexion ^†^	18.8 ± 7.6(38)	21.5 ± 7.7 *	0.353	17.5 ± 7.9(47)	21.9 ± 8.0 *	**0.553**	19.4 ± 7.7(47)	19.9 ± 8.3	0.063
Functional movement screen (scores) ^†^	16.6 ± 3.4	19.9 ± 3.9 *	**0.904**	15.9 ± 3.7	18.8 ± 5.3 *	**0.644**	16.2 ± 3.7	16.8 ± 4.3 ^$,^	0.150

Note: MT, multicomponent training; MFT, multicomponent and flexibility training; CG, control group. ^†^ Interaction between time and group *p* < 0.05. ^‡^ Time effect *p* < 0.05. * *p* < 0.05 in relation to the pre-intervention moment in the same group. ^#^ *p* < 0.05 in relation to the MFT at the same moment. ^$^ *p* < 0.05 in relation to the MT at the same moment. Bold: effect size ≥ 0.50.

**Table 4:** Effect size of postural evaluation (biophotogammetry).

Postural Evaluation	MT	MFT	CG
Anterior view			
Head	⊗	⊗	⊗
Torso	⊗⊗⊗	⊗⊗⊝	⊗⊗⊗
Lower limbs	⊗⊗⊗⊗⊗⊗	⊗⊗⊗⊝⊝⊝	⊗⊗⊗⊗⊗⊗
Posterior view			
Torso	⊗	⊗	⊗
Lower limbs	⊗⊗	⊗⊝	⊗⊝
Right lateral view			
Head	⊗⊗	⊗⊝	⊗⊝
Torso	⊗⊗⊗⊗	⊗⊗⊝⊝	⊗⊗⊗⊝
Lower limbs	⊗⊗	⊗⊝	⊗⊗
Left lateral view			
Head	⊗⊝	⊗⊝	⊗⊗
Torso	⊗⊗⊗⊗	⊗⊗⊝⊝	⊗⊗⊝⊝
Lower limbs	⊗⊝	⊝⊝	⊗⊗
Asymmetries			
Frontal and sagittal planes	⊗⊝	⊝⊝	⊗⊗

Note: MT, multicomponent training; MFT, multicomponent and flexibility training; CG, control group; **⊗**, effect size < 0.50; **⊝**, effect size ≥ 0.50.

## Data Availability

The data presented in this study are available on request from the corresponding author. The data are not publicly available due to consent provided by participants.

## Supplementary Materials

The following are available online at https://www.mdpi.com/article/10.3390/ijerph182010709/s1, Table S1: Postural evaluation (biophotogammetry), anterior view, at the pre and post experimental period moments, Table S2: Postural evaluation (biophotogammetry), posterior view, at the pre and post experimental period moments, Table S3: Postural evaluation (biophotogammetry), right lateral view, at the pre and post experimental period moments, Table S4: Postural evaluation (biophotogammetry), left lateral view, at the pre and post experimental period moments, Table S5: Postural evaluation (biophotogammetry) of the asymmetries of the frontal and sagittal planes at the pre and post experimental period moments.
