# Supplementary material for: Effect of Flexibility Training Associated with Multicomponent Training on Posture and Quality of Movement in Physically Inactive Older Women: A Randomized Study
Source: Int J Environ Res Public Health. 2021 Oct 13;18(20):10709. doi: 10.3390/ijerph182010709 (PMC8536106; doi:10.3390/ijerph182010709)
Supplement: Supplementary file 1 [file ijerph-18-10709-s001.zip › ijerph-1315246-SI.pdf]

International Journal of
Environmental Research and Public Health

MDPI

Table S1: Postural evaluation (biophotogammetry), anterior view, at the pre and post experimental period moments.

	MT (n=52)			MFT (n=43)			CG (n=47)		
	Pre	Post	Effect size	Pre	Post	Effect size	Pre	Post	Effect size
<u>Head</u>									
HHA ( )‡	3.8 ± 6.0	3.0 ± 5.2	-0.143	5.1 ± 5.5	2.9 ± 4.4	-0.444	5.6 ± 5.3	6.0 ± 4.0	0.086
<u>Torso</u>									
HAA ( )‡	4.6 ± 3.5	4.5 ± 3.6	-0.028	4.5 ± 3.6	3.1 ± 2.7	-0.444	4.4 ± 3.4	4.6 ± 2.4	0.069
ASISHA ( )‡	4.2 ± 2.7	3.5 ± 2.6	-0.264	4.3 ± 2.4	2.8 ± 2.4	**-0.625**	4.0 ± 2.6	4.6 ± 2.3	0.245
ASISAA ( )	1.3 ± 5.1	1.0 ± 4.6	-0.062	0.6 ± 5.7	0.3 ± 3.8	-0.063	0.1 ± 5.6	0.3 ± 3.5	0.044
<u>Lower limbs</u>									
RLLFA (º $x10^{-1}$)	0.2 ± 0.2	0.1 ± 0.2	-0.054	0.1 ± 0.2	0.2 ± 0.1	0.063	0.1 ± 0.2	0.2 ± 0.1	0.065
LLLFA (º $x10^{-1}$)	1.0 ± 1.9	1.0 ± 1.6	0.000	0.1 ± 2.1	1.0 ± 1.0	0.058	0.5 ± 2.0	1.0 ± 0.9	0.034
LLD (cm)‡	4.1 ± 3.0	3.4 ± 2.9	-0.237	4.1 ± 4.3	1.8 ± 3.2	**-0.613**	3.2 ± 4.1	3.8 ± 2.4	0.185
TTA (º)‡	3.9 ± 3.0	3.2 ± 1.9	-0.286	2.9 ± 4.1	1.8 ± 2.1	-0.355	2.9 ± 4.0	2.8 ± 1.9	-0.034
RQA (º)‡	17.9 ± 1.3	17.4 ± 1.3	-0.385	17.8 ± 1.6	16.6 ± 1.3	**-0.828**	17.7 ± 1.6	17.6 ± 1.1	-0.074
LQA (º)‡	17.8 ± 2.0	17.1 ± 1.7	-0.378	17.7 ± 2.2	16.6 ± 1.5	**-0.595**	17.6 ± 2.3	17.5 ± 1.5	-0.053

Note: MT, multicomponent training; MFT, multicomponent and flexibility training; CG, control group; HHA, horizontal head alignment; HAA, horizontal acromion alignment; ASISHA anterior superior iliac spine horizontal alignment; ASISAA, anterior superior iliac spine acromial angle; RLLFA, right lower limb frontal angle; LLLFA, left lower limb frontal angle; LLD, limb length difference; TTA, tibial tuberosity alignment; RQA, right Q angle; LQA, left Q angle.

‡ Time effect $p<0.05$.

Bold: effect size ≥ 0.50.

Table S2: Postural evaluation (biophotogammetry), posterior view, at the pre and post experimental period moments.

	MT (n=52)			MFT (n=43)			CG (n=47)		
	Pre	Post	Effect size	Pre	Post	Effect size	Pre	Post	Effect size
Torso									
HSA (cm x10^{-2})‡	5.2 ± 9.1	4.0 ± 7.5	-0.145	6.3 ± 8.7	3.8 ± 6.3	-0.333	6.5 ± 8.7	3.8 ± 6.1	-0.365
Lower limbs									
RLHA (º x10^{-3})‡	10.4 ± 16.2	9.2 ± 11.8	-0.086	9.3 ± 14.2	7.0 ± 10.3	-0.188	9.6 ± 1.2	6.8 ± 9.1	**-0.544**
LLHA (º x10^{-3})‡	15.0 ± 12.6	13.1 ± 8.2	-0.183	15.8 ± 10.2	11.4 ± 7.4	**-0.500**	13.0 ± 8.3	9.6 ± 6.5	-0.459

Note: MT, multicomponent training; MFT, multicomponent and flexibility training; CG, control group; HSA, horizontal scapular asymmetry; RLHA, right leg and hindfoot angle; LLHA, left leg and hindfoot angle.

‡ Time effect $p<0.05$.

Bold: effect size ≥ 0.50

Table S3: Postural evaluation (biophotogammetry), right lateral view, at the pre and post experimental period moments.

	MT (n=52)			MFT (n=43)			CG (n=47)		
	Pre	Post	Effect size	Pre	Post	Effect size	Pre	Post	Effect size
Head									
HAHC7 (º x 10^{-1})†	8.0 ± 0.2	6.0 ± 1.0*	**-3.333**	8.0 ± 0.2	5.0 ± 2.0*$	**-2.727**	8.0 ± 0.2	9.4 ± 1.0$	**2.333**
VAHA (º)‡	0.8 ± 0.7	0.7 ± 1.0	-0.118	0.6 ± 1.1	0.4 ± 0.8	-0.211	0.5 ± 1.1	0.6 ± 0.8	0.105
Torso									
VTA (º)‡	0.2 ± 0.6	0.1 ± 0.5	-0.182	0.3 ± 0.6	0.2 ± 0.3	-0.222	0.3 ± 0.6	0.6 ± 0.3	**0.667**
HA (º)‡	0.5 ± 0.7	0.4 ± 0.6	-0.154	0.5 ± 0.8	0.1 ± 0.5	**-0.615**	0.4 ± 0 .8	0.7 ± 0.5	0.462
VBA(º)	0.4 ± 2.0	0.2 ± 0.9	-0.138	0.5 ± 2.1	0.2 ± 0.9	-0.200	0.5 ± 2.0	0.5 ± 0.9	0.000
HPA (º)‡	5.0 ± 3.1	4.1 ± 2.7	-0.310	4.6 ± 4.0	1.1 ± 2.9	**-1.014**	4.5 ± 4.0	4.9 ± 2.7	0.119
Lower limbs									
AA (º)†	8.3 ± 1.5	7.3 ± 2.7*	-0.476	8.5 ± 2.2	5.4 ± 2.5*$	**-1.319**	8.0 ± 1.4	8.3 ± 2.6	0.150
KA (º x10^{-1})	1.0 ± 1.6	2.0 ± 19.0	0.097	1.0 ± 18.0	2.0 ± 9.0	0.074	0.1 ± 17.0	1.0 ± 9.0	0.069

Note: MT, multicomponent training; MFT, multicomponent and flexibility training; CG, control group; HAHC7, horizontal alignment of the head with the C7; VAHA, vertical alignment of the head with the acromion, VTA, vertical torso alignment; HA, hip alignment; VBA, vertical body alignment; HPA, horizontal pelvic alignment; AA, ankle angle; KA, knee angle.

† Interaction between time and group ($p<0.05$).

‡ Time effect ($p<0.05$).

* $p<0.05$ in relation to the pre-intervention moment in the same group.

Table S4: Postural evaluation (biophotogammetry), left lateral view, at the pre and post experimental period moments.

	MT (n=52)			MFT (n=43)			CG (n=47)		
	Pre	Post	Effect size	Pre	Post	Effect size	Pre	Post	Effect size
Head									
HAHC7 (º)†	8.4 ± 2.7	6.8 ± 1.4*	**0.780**	7.9 ± 2.2	5.3 ± 2.0*$	**1.238**	7.3 ± 2.9	7.2 ± 2.1$	0.040
VAHA (º)‡	0.6 ± 1.4	0.4 ± 1.3	-0.148	0.5 ± 1.2	0.3 ± 1.0*	-0.182	0.4 ± 1.0	0.6 ± 0.7	0.235
Torso									
VTA (º)‡	0.2 ± 0.6	0.1 ± 0.5	-0.182	0.3 ± 0.6	0.2 ± 0.3	-0.222	0.3 ± 0.6	0.6 ± 0.3	**0.667**
HA (º)‡	0.2 ± 0.4	0.2 ± 0.3	0.000	0.4 ± 0.5	0.2 ± 0.3	**-0.500**	0.4 ± 0.4	0.6 ± 0.2	**0.667**
VBA (º)‡	0.9 ± 0.9	0.7 ± 0.7	-0.250	0.6 ± 1.0	0.1 ± 0.7	**-0.588**	0.7 ± 1.0	0.6 ± 0.7	-0.118
HPA (º)	0.1 ± 2.7	0.3 ± 1.3	0.100	0.1 ± 2.9	0.2 ± 1.4	0.047	0.2 ± 2.8	0.2 ± 1.3	0.000
Lower limbs									
AA (º)†	6.4 ± 2.4	5.4 ± 2.5*	-0.408	5.6 ± 2.8	2.7 ± 3.2*$	**-0.967**	5.5 ± 3.7	6.5 ± 2.9$	0.303
KA (º)‡	51.7 ± 20.5	41.6 ± 19.7	**-0.502**	55.1 ± 20.7	32.4 ± 17.3	**-1.195**	53.3 ± 21.8	56.5 ± 13.8	0.180

Note: MT, multicomponent training; MFT, multicomponent and flexibility training; CG, control group; HAHC7, horizontal alignment of the head with the C7; VAHA, vertical alignment of the head with the acromion; VTA, vertical torso alignment; HA, hip alignment; VBA, vertical body alignment; HPA, horizontal pelvic alignment; AA, ankle angle; KA, knee angle.

† Interaction between time and group (p<0.05).

‡ Time effect (p<0.05).

* p<0.05 in relation to the pre-intervention moment in the same group.

$ p<0.05 in relation to the MT at the same moment.

Bold: size effect ≥ 0.50.

Table S5: Postural evaluation (biophotogammetry) of the asymmetries of the frontal and sagittal planes at the pre and post experimental period moments.

	MT (n=52)			MFT (n=43)			CG (n=47)		
	Pre	Post	Effect size	Pre	Post	Effect size	Pre	Post	Effect size
FPA (cm)†	45.8 ± 17.2	39.3 ± 17.3*	-0.377	55.7 ± 20.0	35.6 ± 14.1*$	**-1.179**	57.2 ± 22.4	56.8 ± 20.4	-0.019
SPA (cm)†	34.9 ± 11.0	28.8 ± 10.3*	**-0.573**	38.4 ± 8.7	24.1 ± 9.5*$	**-1.571**	38.1 ± 10.0	39.5 ± 11.9	0.128

Note: MT, multicomponent training; MFT, multicomponent and flexibility training; CG, control group; FPA, frontal plane asymmetry; SPA, sagittal plane asymmetry.

† Interaction between group and time ($p<0.05$).

* $p<0.05$ in relation to the pre-intervention moment in the same group.

$ $p<0.05$ in relation to the MT at the same moment.

Bold: effect size ≥ 0.50.
